# *Inxeba Elinga Phakathi:* The Danger of Mental Health Invisibility and the Role of Social Community Caregiving

**DOI:** 10.3390/ijerph22050786

**Published:** 2025-05-16

**Authors:** Nobuntu Penxa-Matholeni

**Affiliations:** Department of Practical Theology and Missiology, Faculty of Theology, Stellenbosch University, Stellenbosch 7600, South Africa; nobuntu@sun.ac.za

**Keywords:** indigenous methodology, inxeba, mental health, struggles, social community caregiving

## Abstract

The stigma and invisibility surrounding mental health often lead to alienation and reinforce societal misconceptions. This paper examines how the concept of inxeba elingaphakathi (the invisible wound) in isiXhosa encapsulates the emotional and psychological pain frequently overlooked in mental health discussions, particularly among Black South African women. Employing an Indigenous storytelling methodology, the study explores how social community caregiving can illuminate these hidden wounds and challenge prevailing stigma. By analyzing the societal factors shaping mental health perceptions, this research advocates for a culturally grounded approach to healing and belonging.

## 1. Introduction

Mental health remains heavily stigmatized, with silence and invisibility further entrenching this stigma. As Corrigan [1] notes, the stigma surrounding mental illness prevents individuals from pursuing their life goals and accessing critical opportunities. This stigma manifests in three key ways: public stigma, self-stigma, and label avoidance, each profoundly shaping personal aspirations. Addressing these forms of stigma is essential for supporting individuals’ recovery journeys.

Public stigma can be challenged through protest, education, and direct engagement, while self-stigma may be mitigated by fostering group identity, reshaping perceptions of mental illness, and promoting informed choices about disclosure. Segalo [2] highlights how silence plays a crucial role in mental health struggles, particularly for Black South African women. Many of these women find it difficult to speak about the traumatic and dehumanizing experiences endured during apartheid and beyond. Silence, often born from shame and a lack of safe spaces, has normalized suffering, leaving many to carry hidden narratives of pain. This leads to inxeba elinga phakathi—the invisible wound of dehumanization and continuous marginalization, particularly for Black South African women. This wound, often inexpressible in words, reflects an embodied pain shaped by historical and structural violence. Without adequate platforms for expression and healing, many remain trapped in cycles of internalized trauma and exclusion.

This paper positions inxeba elingaphakathi as a conceptual lens to examine how Black South African women navigate mental health struggles amid pervasive stigma. Drawing on Indigenous storytelling methodology, it explores how social community caregiving can serve as a transformative approach to making the invisible wound visible and facilitating collective healing. Indigenous storytelling is not merely a narrative technique but an epistemological framework that affirms cultural knowledge systems and communal approaches to well-being. By centering Indigenous epistemologies within contemporary mental health discourse, this study challenges dominant Western psychiatric paradigms that often reduce mental health to individual pathology, overlooking the broader socio-historical determinants of distress.

Furthermore, this study connects its insights to global mental health frameworks, including the World Health Organization’s (WHO) definitions of mental health and the significance of stigma reduction [3]. By engaging both Indigenous and global perspectives, it advocates for culturally grounded approaches to healing and belonging, recognizing the importance of social determinants of mental health, self-determination, and freedom from discrimination.

At the heart of this study is the concept of inxeba elingaphakathi, which serves as a lens to examine how Black South African women navigate mental health struggles amid pervasive stigma. This analysis is necessary because dominant psychiatric models often fail to account for the historical and social wounds carried by Black South African women, framing mental health narrowly as individual pathology. By overlooking the collective, cultural, and structural roots of distress, these models risk perpetuating exclusion and misdiagnosis. This paper, therefore, offers a socially grounded and epistemologically relevant alternative. This gap has been widely noted in recent scholarship, which highlights the limitations of Western psychiatry in engaging with culturally situated experiences of distress, particularly in African contexts where Indigenous knowledge systems remain vital yet marginalized, Letsoalo et al. [4].

Drawing on Indigenous storytelling methodology, the paper explores how social community caregiving can serve as a transformative approach to making the invisible wound visible and facilitating collective healing. Indigenous storytelling is not merely a narrative technique but an epistemological framework that affirms cultural knowledge systems and communal approaches to well-being. Ultimately, it argues that mental health interventions must be contextually rooted, drawing from local knowledge systems to support sustainable healing practices.

## 2. Self-Location

The positioning of indigenous researchers emphasizes the interplay between lived experiences, cultural backgrounds, and indigenous ways of knowing [5]. In this article, I do more than simply locate myself—I actively acknowledge my biases as a Black South African woman who, like many others, has borne the silence imposed during apartheid and the enduring trauma that accompanies it. Although mental health is traditionally rooted in medical disciplines, I address this topic from the perspective of a pastoral theologian, drawing on the strengths of social community caregiving. My approach emphasizes the sociocultural understanding outlined by De Vincenzo, Stocco, and Modugno [6], which underscores the significance of cultural and communal contexts in knowledge transmission. This perspective allows me to offer support that recognizes the interconnectedness of individuals, communities, and culture in the healing process.

My experiences are deeply embedded in the culture that informs this work. Ukuzithutha, or “self-praise”, as introduced by Chilisa [7], underscores the value of self-knowledge and identity in Black African cultures. Among the amaXhosa, this practice situates the individual within a broader familial and communal context. For example, when I recite my clan names, such as Mxesibe and Nxele, to name just a few, I call upon and awaken the ancestral spirits—the very bones of those who have passed on and those who still live—thereby invoking the full continuum of my paternal and maternal legacies. This act of recitation—affirmed by Chilisa’s assertion that “the definition of self is intertwined with the environment and its inhabitants—people, animals, birds, and vegetation”—fosters a profound sense of belonging, harmony, and healing.

Therefore, I choose to position myself in this article through ukuzithutha, aligning with the indigenous storytelling methodology employed here to facilitate healing. I am the granddaughter of the amaMpondomise, oJola, uZanyiwe, and intombi ezala umama on my maternal side, and the granddaughter of the amaGcina, uSalazi, and intombi ezala utata on my paternal side, Penxa-Matholeni [8]. These ancestral ties anchor my identity. As an amaXhosa woman raised in a village, my individuality thrives in tandem with my community, and these ancestral praises—passed down through generations—serve as a hallmark of this identity [9]. This perspective shapes my interpretation of the text [8] and reinforces my commitment to a culturally grounded, holistic approach to mental health.

## 3. Methodology

The Indigenous storytelling method utilized in this study builds upon a framework I previously proposed for studying pastoral care and counseling. In Black African communities, storytelling is deeply embedded in various forms, including iingoma (traditional songs), umxhentso (traditional dance), iintsomi/izinganekwane (folktales), eziko (stories shared by the fire), ukubetha izandla (clapping of hands), as well as the names of individuals, places, and metaphors. These narrative forms serve as vital tools for preserving, analyzing, and transmitting knowledge while also facilitating socialization. Chilisa [7] emphasizes that Indigenous languages and oral traditions provide critical insights into the lived experiences of Indigenous peoples, reflecting the richness of diverse cultures and epistemologies. This study extends that perspective by foregrounding the role of storytelling in dismantling mental health stigma, particularly through the lens of inxeba elingaphakathi (the invisible wound), acknowledging the cultural and contextual nuances of Black South African women’s experiences.

Indigenous storytelling is not merely a narrative technique but an epistemology—an embodied way of knowing and meaning-making, deeply connected to the land, people, and ancestors. Unlike Western research traditions, which prioritize written texts and empirical objectivity, Indigenous epistemologies value oral traditions and communal knowledge passed down through generations. In Black African traditions, knowledge is not an individual possession; rather, it is relational and collectively held. My inclusion of clan names, songs, rituals, and metaphors in Indigenous storytelling is intentional—it serves as a bridge between past, present, and future.

Western methodologies often separate mind and body, whereas Indigenous knowledge systems recognize embodied knowing. The physical acts of clapping hands, singing, or dancing in Indigenous storytelling are not merely performative; they are integral to knowledge transmission, carrying memory, spirituality, and connection. The land itself is a participant in knowledge-making, as stories, names, and rituals are tied to specific places and ancestral presences.

Using storytelling as an Indigenous research method is an act of decolonization—it disrupts dominant narratives that have historically erased Indigenous voices. This methodology is particularly valuable in examining mental health stigma, as it addresses the emotional and psychological aspects of healing. Singing and the invocation of clan names remind those experiencing mental distress that support extends beyond the immediate community; it includes the presence of ancestors, a vital source of belonging and affirmation often overlooked in conventional research methods. Furthermore, this methodology acknowledges healing as a critical part of knowledge production, making it particularly relevant in studies related to mental health and identity.

## 4. Mental Health

The “wound that is inside” refers to a form of invisible emotional or psychological trauma that deeply affects an individual, even though it is not immediately visible or physically manifest. In medical terms, this kind of wound can be seen as a form of psychological injury or mental health trauma, often associated with long-term stress, neglect, abuse, or experiences of dehumanization.

Medically, this “invisible wound” can be related to post-traumatic stress disorder (PTSD), depression, anxiety disorders, or other mental health conditions that result from long-term emotional harm. These conditions often involve disturbances in how a person processes their experiences, memories, and emotions. In this context, the wound is internal, affecting the mental, emotional, and even physical health of the individual.

The wound may not be visible on the surface, but it can manifest in various ways, such as through feelings of shame, isolation, numbness, or emotional reactivity. The psychological effects of such wounds may lead to difficulties in relationships, a sense of being disconnected from one’s identity, and struggles with self-worth.

### 4.1. Inxeba Elingaphakathi

Transitioning to inxeba (the invisible wound), it is important to acknowledge that in many African cultures, trauma is not merely an individual experience but also a collective one. The concept of ‘inxeba’ embodies a deep, invisible wound caused by systemic, historical, or cultural harm, such as dehumanization, gender-based violence (GBV), and the marginalization of Black (South) Africans during colonization and apartheid. This wound is often carried by communities across generations, transmitted through unspoken experiences and inherited trauma. Inxeba reflects a profound emotional, psychological, and spiritual scar that continues to affect those who bear it, even when it is not immediately visible.

An example of these deep generational wounds is evident in the narratives of the so-called “marginalized”. In these contexts, inxeba becomes a shared and familiar burden within communities, families, and even workplaces. There is often an unspoken expectation to keep moving forward because “everybody is going on”. These narratives remain unwritten—they originate in life itself rather than in formal data collection. As Okure [10] aptly states, “First was the life, not the book. Only in life does the book become life”.

The narratives presented below emerged from conversations about mental health that took place in various contexts and settings. They were not gathered through structured data collection but rather reflect the lived experiences of individuals. They serve as life itself, embodying the organic transmission of deep, personal, and collective wounds:

First narrative:

“*In our community, we cannot afford to be depressed. We must put food on the table, so we just carry on with it*”.

Second narrative:

“*After my husband died during COVID-19, I became depressed and reported it at work. However, I wish I hadn’t, because it was used against me. I was told I couldn’t be promoted because I was ‘mentally unstable.’ Since then, whenever I need to consult my psychologist for my mental health, I just take leave. I don’t share anything at work anymore*”.

### 4.2. Theorizing Inxeba Elingaphakathi: African Women’s Experiences of Trauma and Resilience

Starting with a theorization of inxeba ensures that the subsequent analysis of narratives is anchored in a well-articulated framework, enabling a richer exploration of how indigenous epistemologies shape the experience and expression of mental health within Black South African communities. This approach offers conceptual clarity, ensuring that inxeba elingaphakathi is understood not merely as a metaphor but as a lived reality, deeply embedded in historical and communal memory. It also situates the narratives within a broader cultural and epistemological landscape, allowing for a more nuanced reading of mental health as a shared, collective burden rather than an individualized struggle.

In the context of amaXhosa culture, the concept of inxeba can be understood both literally and figuratively. Literally, it refers to a physical wound caused by an intentional act of harm—visible and requiring immediate attention. However, inxeba elingaphakathi describes an internal wound inflicted by individual or systemic harm. As the phrase implies, this wound is invisible, yet its impact runs deep. It lingers beneath the surface, unacknowledged yet shaping how people navigate their realities. The silence surrounding these wounds often reinforces their depth, making them all the more difficult to heal.

To illustrate, when someone is re-traumatized by an event, a song, or a memory that triggers the pain of inxeba elingaphakathi (invisible wound), they might say “Lengoma okanye esiganeko sindivusela amanxeba,” meaning, “This song or event is reopening my wounds”. This expression captures how profoundly such experiences can resonate, evoking pain that feels as fresh as the initial harm. Interestingly, the concept can also carry a positive connotation. For instance, the phrase “Yatsho emanxebeni,” meaning “It struck in the wounds”, is used to describe something that deeply moves or provides inner solace.

Unlike a physical wound, inxeba elingaphakathi—the hidden or internal wound—bleeds metaphorically. Its healing is not straightforward, as the wound may stop “bleeding” yet remain tender, vulnerable to being reopened by new triggers or unresolved trauma.

This serves as the point of departure for analyzing and interpreting the narratives above. Let me begin with the first story, which I reflected on with sadness in my heart. It seems that, for some of us, depression is considered a luxury. By this, I mean that the space and time required to process depression is a privilege—one not afforded to those in communities where survival demands constant motion. In these contexts, where every day is filled with the urgency of meeting basic needs, taking time to tend to mental health feels more like an indulgence than a right. The harsh realities of daily life leave little room for reflection or emotional respite, reinforcing the notion that prioritizing one’s mental well-being is a luxury only a few can afford. The stakes are high for those who do not keep moving. What is at stake? In this case, the storyteller numbs their depression because addressing it comes with significant consequences. This leaves no space to talk about it or even acknowledge its existence.

Similarly, in the second story, the narrators numb their depression at work and attempt to carry on. This indicates that when depression reaches its peak in both stories, many questions remain unanswered. The sad part of the second story is that it echoes the difficult times of the HIV and AIDS crisis, when it was not easy to disclose one’s condition. The stigma surrounding both mental health and HIV/AIDS is not only deeply personal but also historically and culturally embedded, making it even harder for individuals to seek help. The stigma surrounding mental health, like that of HIV and AIDS, often piles on—each layer making the struggle more invisible and harder to address. Even more distressing is depression’s invisibility—it cannot be seen with the naked eye. The symptoms cannot be observed because depression is a psychological illness rather than a physical one. The danger lies in the silent struggle, where individuals carry on without anyone knowing what they are enduring. In contrast, HIV and AIDS often present physical symptoms that can be observed. However, the striking similarity between HIV and AIDS and mental health lies in the pervasive stigma that surrounds both, compounding the challenges faced by those affected. This stigma, which is both deeply ingrained and cultural, silences the pain and isolates the individual, preventing open conversation and support.

### 4.3. The Burden of Artificial Horns: Navigating Survival and Disguise

The storytellers in these narratives are compelled to wear what Masenya [11] calls “artificial horns”. To provide context for Masenya’s metaphor, let me briefly explore its origins and how it connects to the experiences of the two narrators. Masenya [11] tells the story of a cow who struggles with insecurities about the appearance of her horns, enduring relentless ridicule from other animals who deem her unattractive. Succumbing to societal pressure and the stigma that labels her as inferior because of her natural horns, the cow undergoes reconstructive surgery, choosing horns that she believes will be more acceptable—those of a merino sheep. This metaphor highlights the internalization of societal judgment and the pressure to conform to external standards, even at the expense of one’s true self. For the narrators, the stigma surrounding their mental health, much like the cow’s artificial horns, forces them to hide their struggles, donning masks that align with what society deems “normal” or acceptable.

Linking the description of the cow to the two narratives, both storytellers metaphorically opt for “reconstructive surgery” to avoid being identified as sufferers of depression. One storyteller chooses to wear these “artificial horns” to avoid being labeled as mentally unstable in the workplace. The other adopts them because they perceive depression as a luxury not afforded to those in their community—societal norms dictate that “everybody wears these artificial horns”. In both cases, the community, driven by survival instincts, has made these “artificial horns” the norm. For one storyteller, this expectation is found in the workplace; for the other, it permeates their community. Both environments have grown accustomed to the weight and discomfort of these horns, which I would describe as disguises. However, as Masenya suggests, these artificial horns ultimately fail to fulfill the original function of the natural ones.

Our storytellers live in survival mode, a state of profound disconnection from their surroundings, their bodies, and their inner selves. In this disconnection, inxeba elingaphakathi (the hidden wound) persists and, at times, deepens. Tragically, this disconnection can culminate in a suicide event that forces society to ask questions, often judgmental ones, but only when it is already too late. Confronting the threat of death and an acute awareness of mortality often leads to an existential hardness—the unbearable discomfort of simply being. This paradox, the tension between the struggle for survival and the burden of existence, underscores the urgent need for a fresh, illuminating perspective to navigate the complexities of life [12].

## 5. Social Community Caregiving and Indigenous Storytelling as Hosts and Vehicles for Healing

In essence, the storytellers experience displacement. Without a safe space for humane dwelling, they endure total dislocation [12]. This dislocation is multilayered, stripping them of stability and leading to psychological, physical, and spiritual displacement, which culminates in extreme anxiety. To contextualize this dislocation, many non-white South Africans were physically uprooted and relocated during apartheid, moved according to their perceived status under the apartheid regime. Specifically, Black South Africans were forcibly removed from their land and separated from white communities because they were deemed “not good enough” to occupy desirable spaces. Other racial groups were placed in areas considered more favorable than those designated for Black South Africans, who were relegated to the bottom of the hierarchy [13]. This removal was not merely physical; it was also spiritual, as the land represents more than just physical space.

Louw [12] asserts that human displacement is an existential, social, and cultural phenomenon. It highlights the gap between safe spaces that promote human well-being and unsafe spaces marked by inhumane conditions. These conditions are often shaped by intimidating experiences, personal and relational disruptions, and environmental upheavals. To become displaced implies being ousted or dislodged. One experiences not merely a loss of place but also degradation in status, position, and dignity. It even points to an implosion of human identity and destruction of a sense of unique personal identity.

The social community caregiving approach, as advocated by pastoral theologian Penxa-Matholeni [8], embraces all aspects of life, including social ills, community connectedness, and the political and cultural context. This holistic approach is what I refer to as “township theology”. If we are to use the words “God” and “Bible” in our care for one another within the context of mental health, we must recognize that a God without a heart is nothing more than an idol.

What I aim to convey aligns with what Louw [12] describes as a theology of the intestine—a form of care that originates deep within, from the depths of one’s being, not as artificial or surface-level care. This resonates with Hammarskjöld (cited in Louw) [12], who critiques superficial interactions: “To be ‘sociable’—to talk because convention forbids silence, to rub against one another to create the illusion of intimacy and contact”. It also recalls Milan Kundera’s poignant observation: “There is nothing heavier than compassion. Not even one’s own pain weighs so heavy as the pain one feels with someone for someone, a pain intensified by the imagination and prolonged by a hundred echoes”.

This is solidarity in pain—genuine caregiving that emerges from deep empathy and connection, the kind that ought to feel weighty. It is care that is imagined and enacted through solidarity, rooted in the very depths of our shared humanity.

### 5.1. Healing Through Social Community Caregiving and Indigenous Stories

The social community caregiving approach operates spontaneously, meeting people where they are in their unique circumstances. I refer to this space as endleleni (along the road). I will return to this concept later to explore the spontaneity and transformative potential of social community caregiving in greater detail.

Dube [14] observes that the margins are where one encounters God; it is in the messiness of life and at the edges of society where God is grasped. Social community caregiving emerges during disorderly times, in the chaos and marginal spaces of life. This form of caregiving is deeply embedded not only in everyday experiences but also in ritual practices such as imbeleko (the ceremony of introducing a newborn to both the living and the ancestors) and utsiki (the ritual of welcoming a newlywed into the family and ancestral realm), to name just a few. Moreover, as Penxa-Matholeni [13] explains, it is multi-layered, spontaneous, and complex, intricately woven into the fabric of culture and the daily struggles of Black Africans.

Elsewhere, I argue that spontaneity in caregiving unfolds within relationships [8]. Social community caregiving embodies the anxieties and lived realities of Black Africans. Louw [13] underscores this point, stating, “If pastoral caregiving does not relate to commonality, communality, connectedness, the everyday struggles, and experiences of humanity for social justice, cannot appeal to black Africans”. This is an opportune moment to reintroduce the concept of endleleni. Endleleni represents resilience, bravery, and strength, not as a destination, but as the experience of the not-yet-home. It is rooted in the following isiXhosa proverb: “Amaqobokazana angalala endleleni yazini kunyembelekile”. Amaqobokazana means “young maidens”. They are referred to as amaqobokazana, not because of their age, but because of the significance of the mission they are undertaking. When these young maidens sleep endleleni (along the road), something of great value is at stake, which is why they are cautioned: “Balala be bambe umkhonto ngobukhali” (“they sleep holding the spear with the sharp edge”) [15].

I propose that the metaphor of endleleni does not signify a destination but rather a journey that invites all fellow travelers to solidarity, particularly with those struggling with mental health. It encourages us to look beyond our current reality toward an unknown, yet hopeful, reality. This metaphor places us in a migratory position, urging us to navigate endleleni together in solidarity while collectively seeking solutions to the challenges we face.

### 5.2. Stories That Heal: An Indigenous Storytelling Perspective

I would like to take a moment to emphasize the diverse forms encompassed by indigenous storytelling methodology. Penxa-Matholeni [8] underscores that, within the Black African context, storytelling weaves together a rich tapestry of cultural expressions. This methodology includes iingoma (traditional songs), umxhentso (traditional dance), iintsomi/inganekwane (mythical tales), clan names, handclapping, the naming of children, place names, rituals, proverbs, and metaphors. In this section, I turn my attention to the transformative role of song (iingoma) in fostering mental health and holistic healing.

Penxa-Matholeni [16] argues that singing communicates in a profoundly unique way, distinct from speeches or sermons. It engages the soul, mind, and body, creating a powerful medium for expression and connection. In the Black African context, singing transcends immediate audiences, weaving bonds with the community, nature, animals, birds, one another, and the divine or ancestors.

Umxhentso (traditional dance) complements this by allowing individuals to physically embody the song’s rhythm, deepening its impact. Together, these elements nurture not only the healing of the soul and spirit but also contribute to physical restoration. During apartheid, songs were not merely acts of resistance or expressions of lamentation—they carried the resonance of ancestral strength and reached toward the divine, offering solace and a collective sense of empowerment. This interplay between song, movement, and connection highlights the significance of cultural practices in addressing mental and emotional well-being.

I will explore the role of songs within the context of mental health. Before exploring the interpretation of the song’s meaning, it is essential to acknowledge that meaning is a social construct. The world does not inherently possess meaning but requires us to imbue it with significance. A text, practice, or event does not originate with meaning; instead, it provides a platform where various interpretations can emerge and coexist.

Because different meanings can be ascribed to the same text, practice, or event, meaning becomes a dynamic and contested space. It serves as both a site of assimilation and resistance—a terrain where hegemonic ideas may be affirmed or challenged. This underscores the potential of music to be imbued with varied meanings, depending on context and purpose. For someone struggling with mental health, the need for belonging is paramount. The stigma surrounding mental illness often fosters a profound sense of isolation and displacement [17]. These feelings of isolation are linked to a deterioration in mental well-being, as those who are marginalized struggle to access social support [17]. These lyrics offer a powerful counter-narrative, restoring a sense of belonging and hope. What one person perceives in a song may differ significantly from another’s interpretation, as our understanding is shaped by our unique experiences and perspectives. With this framework in mind, I will offer an interpretation of the song’s meaning from my own perspective.

Below are the lyrics of the song I will analyze: “Jerusalema (feat. Nomcebo Zikode)” by Master KG.
Jerusalem ikhaya lami, (Jerusalem is my home)Ngilondoloze, (Guard me)Uhambe nami, (walk with me)Zungangishiyi lana, (Do not leave me here)Jerusalema ikhaya lami, (Jerusalem is my home)Ngilondoloze, (Guard me)Uhambe nami, (Walk with me)Zungangishiyi lana, (Do not leave me here)Jerusalema ikhaya lami, (Jerusalema is my home)Ngilondoloze, (Guard me)Uhambe nami, (Walk with me)Zungangishiyi lana, (Do not leave me here)Ndawo yami ayikho lana, (My place is not here)Mbuso wami awukho lana, (My kingdom is not here)Ngilondoloze, (Guard me)Zuhambe nami, (Walk with me)Ngilondoloze, (Guard me)Ngilondoloze, (Guard me)Ngilondoloze, (Guard me)Zungangishiyi lana, (Do not leave me here)Jerusalema ikhaya lami, (Jerusalem is my home)Ngilondoloze, (Guard me)Uhambe nami, (walk with me)Zungangishiyi lana, (Do not leave here)Ngilondoloze, (Guard me)Ngilondoloze, (Guard me)Ngilondoloze, (Guard me)

This song became a viral sensation and a reflective anthem in South Africa as the world faced the challenges of COVID-19. On 16 September 2020, President Cyril Ramaphosa, in an address to the nation, encouraged everyone to use Heritage Day as an opportunity for family time, to reflect on the difficult journey the nation had endured, to honor those who had lost their lives, and to quietly celebrate the remarkable and diverse heritage of South Africa [18]. He also invited the country to participate in the *Jerusalema* dance challenge, adding a unifying and uplifting element to the occasion.

The President’s statement put the song on the map; it is safe to say that some South Africans were unaware of the song until then. The collective act of dancing and singing to the song fosters a sense of healing. From a mental health perspective, dancing brings the group into the rhythm of life, as participants not only sing but also reflect on the lyrics. Social community caregiving plays a pivotal role in facilitating this connection, strengthening the bonds within the group and extending to the wider relationships they share.

In my interpretation of the song from a mental health perspective, I focus on specific lyrics that carry profound meaning for individuals navigating the complexities of mental health. These lyrics include the following:
Jerusalema ikhaya lami (Jerusalem is my home)Ngilondoloze (Guard me)Uhambe nami (Walk with me)Zungangishiyi lana (Do not leave me here)Ndawo yami ayikho lana (My place is not here)Mbuso wami awukho lana (My kingdom is not here)Jerusalema ikhaya lami (Jerusalem is my home)

For someone struggling with mental health, the need for belonging is paramount. The stigma surrounding mental illness often fosters a profound sense of isolation and displacement. This is where music, particularly lyrics like ‘Jerusalema ikhaya lami’ (Jerusalem is my home), offers a powerful counter-narrative—restoring a sense of belonging and hope. These words speak to the deep human need for connection and rootedness. For someone feeling displaced by the weight of mental illness, they reaffirm the promise of a home—an emotional and spiritual space where they are accepted, loved, and at peace. It serves as a reminder that they are not forgotten and that they belong somewhere sacred and nurturing”.

“Jerusalema ikhaya lami” (Jerusalem is my home): This line speaks to the deep human need for connection and rootedness. For someone feeling displaced by the weight of mental illness, it reaffirms the promise of a home—an emotional and spiritual space where they are accepted, loved, and at peace. It serves as a reminder that they are not forgotten and that they belong somewhere sacred and nurturing.

“Ngilondoloze” (Guard me): The word Ngilondoloze carries a depth of meaning that extends beyond mere guarding. It conveys the act of being tenderly shielded, cradled, and enveloped in a protective embrace. It evokes an image of being cocooned with care, safeguarded in both body and spirit, and nurtured toward healing and wholeness. For someone battling depression or anxiety, ngilondoloze becomes a plea for protection—not only from external threats but also from the inner turmoil that weighs heavily on them. It is a cry for intentional, loving care and the hope of being preserved through the storm.

“Uhambe nami” (Walk with me): This phrase is more than an invitation for physical companionship; it symbolizes a deeper yearning for connection and shared presence. It embodies the need to be seen, acknowledged, and to have one’s journey recognized and supported—not just by others but also by nature, animals, God, and ancestors. For someone battling depression, it conveys the profound comfort of having support in life’s loneliest and most shadowy places, including the dark recesses of the mind where suicidal thoughts may linger. It is a heartfelt plea for solidarity and accompaniment through the unseen struggles, a request not to walk the journey alone.

“Zungangishiyi lana” (Do not leave me here): This line embodies a desperate plea against abandonment in the depths of despair. For someone enduring the darkness of depression—inxeba elingaphakathi (the wound within)—it becomes a heartfelt cry for rescue from emotional and mental anguish. It speaks to the deep yearning to be drawn out of isolation and despair and into a place of light, connection, and healing.

“Ndawo yami ayikho lana” (My place is not here): This lyric is a bold declaration of hope. It asserts that one’s current state of suffering, darkness, or mental anguish is not their ultimate destination. It reflects a refusal to be defined by pain or depression, embodying the belief that there is something greater beyond the present struggle.

“Mbuso wami awukho lana” (My kingdom is not here): This line speaks to a higher accountability and identity that transcends the current moment. It reminds the listener that their worth and purpose are not confined to their suffering. Their “kingdom” lies beyond the present reality, offering a sense of hope, purpose, and liberation from the chains of mental anguish.

In these lyrics, the song transforms into a profound anthem of hope and resilience for those facing the challenges of inxeba elingaphakathi. It restores a sense of belonging, offers the promise of protection, and inspires the belief that healing and peace are within reach.

These healing stories, including songs, can be easily facilitated by community caregiving that is inspired by the context and immediate needs of the people. Resilience, hope, a sense of belonging, and healing occur in spaces conducive to accommodating the diverse indigenous stories that bring healing to individuals.

## 6. Conclusions

In conclusion, the exploration of inxeba elingaphakathi—an invisible wound that manifests in emotional, psychological, and spiritual trauma—illuminates a critical understanding of the deep-seated, collective suffering carried across generations. Rooted in African cultural narratives, this wound underscores the silent struggles faced by individuals and communities, often driven by systemic and historical oppression, such as GBV, marginalization, and the dehumanization of Black South Africans. The shared narratives reveal the paradox of survival and the disconnection from one’s true self, as individuals endure the weight of an invisible wound, numbing their pain to avoid stigma.

The concept of “artificial horns”, as illustrated by Masenya, resonates strongly in these stories, where individuals mask their trauma, suppressing their mental health struggles to survive within societal expectations. Yet, this survival mode often perpetuates a cycle of dislocation and existential discomfort, which, if left unaddressed, can lead to irreversible outcomes, such as suicide.

However, in this paper, we also observe pathways to healing. Social community caregiving, rooted in African indigenous wisdom, offers a form of care that is spontaneous, relational, and deeply connected to the lived experiences of individuals. It is within the spaces of community, culture, and spiritual practice that the healing of inxeba elingaphakathi can begin. The metaphor of endleleni invites us to embrace resilience, solidarity, and collective action, walking together in understanding as we confront the complexities of mental health.

Indigenous storytelling, exemplified through song, dance, and rituals, holds immense potential for healing. The healing power of song, such as the spiritual resonance of Jerusalema, draws individuals and communities into connection, facilitating a collective healing that transcends individual pain. Through storytelling, we can reframe trauma and offer pathways to restoration and empowerment, turning the invisible wound into a source of shared strength and resilience.

Ultimately, as we continue to navigate the invisible wounds of our communities, we must center our care in the cultural, social, and spiritual practices that resonate with the people we seek to help. Only through understanding, solidarity, and the embrace of indigenous healing traditions can we begin to address the complexities of mental health in a way that honors the dignity and experiences of those who carry these hidden wounds.

## Data Availability

No new data were created or analyzed in this study. Data sharing is not applicable to this article.
